# Camel hump: composition, bioactivities, and multifunctional sustainable applications

**DOI:** 10.3389/fnut.2026.1869754

**Published:** 2026-06-25

**Authors:** Muyassar Yasen, Xierzhati Aihaiti, Sabahat Ablimit, Zheng Ren, Ajiguli Waisiding, Mubarak Iminjan, Yuan Ma

**Affiliations:** 1 College of Pharmacy, Xinjiang Medical University, Urumqi, Xinjiang, China; 2 The Sixth Affiliated Hospital of Xinjiang Medical University, Urumqi, China

**Keywords:** camel hump, chemical composition, multiple applications, multiple bioactivities, sustainable utilization

## Abstract

Camel hump, a valuable livestock by-product, has gained increasing attention for its unique chemical composition and diverse biological activities. Given its promising potential in traditional medicine and modern industrial applications, this review first systematically summarizes the chemical composition of camel hump and examines key influencing factors, including species, breed, sex, age, and physiological status. We then highlight its notable application potential in cosmetics, supported by strong antioxidant and anti-aging enzymatic activities. We further outline its innovative uses in the food industry, materials science, and leather processing, with emphasis on its unique merits in improving fatty acid profiles of meat products and developing biomimetic thermal management materials. Finally, we discuss the importance of establishing standardized raw material systems and conducting rigorous preclinical studies to provide a solid scientific foundation for the in-depth and sustainable utilization of camel hump. By comprehensively reviewing the material basis, bioactivities, and multifunctional applications of camel hump, this review aims to promote valorization of this distinctive natural by-product, advance the circular economy, facilitate regional technological innovation, and support sustainable management of animal resources.

## Graphical abstract

**Figure:**
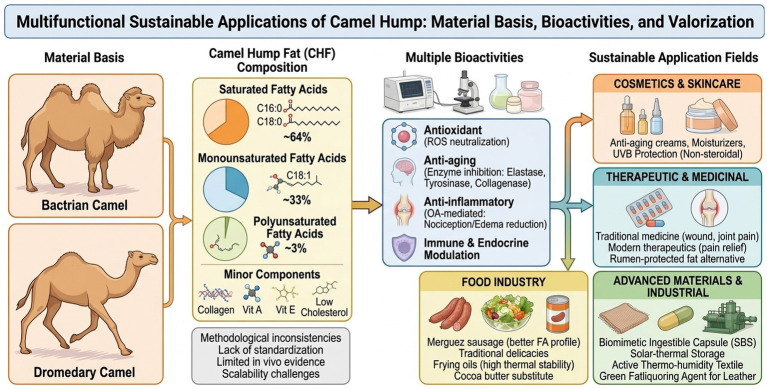

## 1 Introduction

Camels originated on the North American continent and were domesticated by semi-nomadicnomadic tribes in southern Arabia around 4,000 years ago. They were primarily used for transportation and labor, rather than as a source of meat, milk, or clothing (1). The family Camelidae comprises two domesticated species: dromedaries and Bactrian camels (*Camelus bactrianus*). Dromedaries primarily inhabit the hot, arid countries of Africa, the Middle East, and South Asia, while Bactrian camels are widely distributed across Central Asia and China (2, 3). Dromedaries far outnumber Bactrians, constituting approximately 90% of the genus *Camelus*.

Camels primarily inhabit arid desert regions with sparse vegetation and dry air, exhibiting exceptional tolerance to extreme heat, solar radiation, water scarcity, rugged terrain, and barren vegetation. Anatomical studies confirm that the fat stored in their humps enables camels to withstand extreme environments such as scorching heat, bitter cold, sandstorms, and prolonged hunger and thirst. These characteristics are closely linked to unique lipid metabolism mechanisms (4, 5). Typically, animal fat is categorized into two types: storage fat and tissue fat. The fat within the hump belongs to storage fat, located as subcutaneous fat beneath the broad dorsal muscles on the camel’s back. It accounts for approximately 30% of the camel’s total carcass weight, representing the largest proportion of fat. Camel hump fat (CHF) is rich in nutrients and functional factors, contains a diverse array of fatty acids, and has low cholesterol content, making it of notable economic value (6).

China has a history of camel husbandry spanning over 3,000 years and is one of the world’s primary regions for Bactrian camels. Currently, China has a population of over 330,000 camels, primarily distributed in the northwestern regions such as Xinjiang, Inner Mongolia, Gansu, and Qinghai (7). The Bactrian camel is a vital economic animal in China’s northwestern desert regions, renowned for its tolerance of extreme environments and a robust immune system. It provides herders with diverse livestock products including milk and meat. Based on geographic distribution, body size, and appearance, Bactrian camels can be classified into the Junggar Bactrian camel of northern Xinjiang and the Tarim Bactrian camel of southern Xinjiang. Among these, the Junggar dairy camel, as the primary milk-producing breed, exhibits superior lactation performance. In northern Xinjiang, Junggar Bactrian camels are predominantly raised through traditional grazing methods, fostering strong immunity against various pathogens. Southern Xinjiang suffers from scarce pasture resources, necessitating supplementary feeding through pen-based farming. Camel digestion relies heavily on rumen microbial fermentation, with fecal microbial composition exhibiting seasonal dynamics: microbial diversity increases during warm seasons, while fiber-degrading bacteria become more abundant in cold seasons. Under identical environmental conditions, the microbial diversity of adult female camels is significantly higher than that of lactating young camels (8).

## 2 Camel hump

### 2.1 Histological function of the hump

The hump is a peak-shaped protrusion formed by the accumulation of subcutaneous fat on the back, covered by skin and a thin layer of connective tissue membrane. Connective tissue extends into the fat, dividing it into lobes and lobules of varying sizes. The hump’s adipose tissue consists of typical unilocular fat cells—large, polygonal cells containing a single large lipid droplet within their cytoplasm, with a flattened oval nucleus positioned at the cell periphery. The hump serves as a major energy reservoir. When camels cannot obtain food, the fat within the hump can be broken down into nutrients required by the body. The oxidation of fat within the hump produces metabolic water, supplying the camel’s vital functions. The concentrated fat mass also aids in maintaining the camel’s constant body temperature.

### 2.2 Chemical composition of the hump

#### 2.2.1 Basic chemical composition of hump tissue

CHF primarily contains saturated fatty acids (63.40%) and monounsaturated fatty acids (34.68%), along with a small amount of polyunsaturated fatty acids (1.92%) (9).

According to research by Wuyun et al., the primary chemical composition of the humps of Alashan Bactrian camels (*Camelus bactrianus*) is as follows (based on wet weight): crude fat (90.64%), moisture (7.26%), protein (2.91%), ash (0.208%), and collagen (2.13%); Its fat-soluble vitamin and cholesterol contents are vitamin A (0.56 mg/100 g), vitamin E (1.94 mg/100 g), and cholesterol (42.33 mg/100 g). Fatty acid composition analysis indicates that camel fat contains a rich variety of fatty acids, primarily comprising palmitic acid, stearic acid, oleic acid, palmitoleic acid, and myristic acid, with contents of 33.5, 22.3, 30.91, 4.22, and 6.87%, respectively. It also contains various unsaturated fatty acids such as linoleic acid, linolenic acid, arachidonic acid, and eicosapentaenoic acid (EPA). The total unsaturated fatty acid content is approximately 40%, making it a high-quality lipid resource (10).

Sahraoui et al. conducted a fatty acid composition analysis of hump fat from Algerian camels (*Camelus dromedarius*), revealing the following detailed fatty acid profile: Saturated fatty acids (SFA) constituted 64.4% of total fatty acids (by weight), while monounsaturated fatty acids (MUFA) and polyunsaturated fatty acids (PUFA) accounted for 33.1 and 2.5%, respectively. The primary saturated fatty acids, palmitic acid and stearic acid, constituted 49.6 and 38.8% of SFAs, respectively (representing 31.5 and 25.5% of total fatty acids). Unsaturated fatty acids (UFAs) were predominantly oleic acid, constituting 78.1% of MUFAs (25.9% of total fatty acids); linoleic acid accounted for 88.5% of omega-6 fatty acids (1.17% of total fatty acids); and linolenic acid comprised 63.9% of omega-3 fatty acids (0.42% of total fatty acids). UFA/SFA ratio was 0.039 (i.e., PUFA ÷ SFA = 2.5 ÷ 64.4), and the ω6/ω3 ratio was 2.81 (Table 1). Female individuals exhibited significantly higher SFA and MUFA content, while ω6, ω3, conjugated linoleic acid (CLA), PUFA content, the PUFA/SFA ratio, and the ω6/ω3 ratio showed no significant differences between sexes. Analysis by breed revealed that the Sahraoui breed exhibited higher levels of MUFA, PUFA, omega-6 fatty acids, and omega-6/omega-3 ratio, while the Tergui breed had a relatively higher proportion of SFA (11).

**Table 1:** Comparison of fatty acid composition in *Camelus dromedarius* and *Camelus bactrianus* hump camels (percentage of total fatty acids).

Fatty acid indicators	*Camelus bactrianus*		*Camelus dromedarius*
	Fore hump	Rear hump	
SFA	**65.9 ± 7.91**	**63.63 ± 3.51**	**64.4**
Myristic acid (C14:0)	7.59 ± 2.03	6.41 ± 0.28	–
Palmitic acid (C16:0)	33.57 ± 0.42	35.11 ± 1.59	31.5
Stearic acid (C18:0)	22.18 ± 4.43	19.58 ± 1.40	25.5
MUFA	**30.23 ± 6.67**	**32.72 ± 1.41**	**33.1**
Palmitoleic acid (C16:1)	4.67 ± 0.96	3.77 ± 0.26	–
Oleic acid (C18:1)	25.04 ± 5.44	28.58 ± 1.00	25.9
PUFA	**3.59 ± 0.47**	**3.22 ± 0.24**	**2.5**
Linoleic acid (C18:2 ω-6)	2.19 ± 0.22	1.92 ± 0.09	1.17
α-Linolenic acid (C18:3 ω-3)	1.22 ± 0.03	1.13 ± 0.02	0.42
Others			
PUFA/SFA	–	–	0.039
ω6/ω3	–	–	2.81

^*^Bactrian camel data are presented as mean ± standard deviation (*n* = 3 per hump position; data from (10) and original analysis). Dromedary camel data are mean values from Sahraoui et al. (11); standard deviations were not reported in the original study. “–” indicates not detected or not reported. Bold values represent the total content of each fatty acid category (SFA, MUFA, PUFA) as reported in the original sources; non-bold values represent selected major individual fatty acids within each category and may not sum exactly to the bold total, as minor or unreported components are not listed individually.

#### 2.2.2 Physicochemical properties of camel oil

The resulting hump oil is a white solid at room temperature with a characteristic mutton odor. Its fundamental physicochemical properties are as follows: density 0.9105 g/mL, melting point 48.19 °C, iodine value 37.83 g I_2_/100 g, saponification value 201.14 mg KOH/g, acid value 0.63 mg KOH/g, peroxide value 2.38 mmol/kg (12).

#### 2.2.3 Key factors affecting hump components

Analysis of variance (ANOVA) confirmed that camel age significantly affected both the nutritional composition of hump tissue and the physicochemical properties of hump fat (*p* < 0.05) (13). Pairwise comparisons between the 5–7-year-old (young *n* = 13) and 8–14-year-old (adult *n* = 14) groups reveal distinct age-related patterns. Younger camels exhibited significantly higher moisture content (mean: 7.26% vs. 6.77%, *p* < 0.05) and protein content (fore hump: 3.20% vs. 2.75%, *p* < 0.01), along with higher iodine values (38.84 vs. 36.69 g I_2_/100 g, *p* < 0.01), indicative of a greater degree of fatty acid unsaturation. In contrast, adult camels showed significantly higher crude fat content (mean: 91.72% vs. 89.96%, *p* < 0.01) and, in the fore hump, elevated acid values (0.73 vs. 0.49 mg KOH/g, *p* < 0.01).

Compared with age, the effects of hump location (front vs. rear) and sex on these parameters are relatively minor. No statistically significant differences (*p* > 0.05) were observed between front and rear humps, or between sexes, for most nutritional and physicochemical indicators (13).

It is important to note that these findings are based on a comparison of two discrete age groups and may not capture continuous age-related trends. Furthermore, the original study did not report full ANOVA tables (including F-statistics and degrees of freedom); only significance levels and mean values with standard deviations were provided. Consequently, these conclusions are presented with these methodological limitations explicitly acknowledged. Future studies with larger cohorts and more complete statistical reporting are needed to confirm these findings.

#### 2.2.4 Comparative nutritional properties of camel fat

According to existing literature data, the major fatty acids in edible beef tallow are palmitic acid (26.43%), stearic acid (19.25%), and oleic acid (30.20%); the essential fatty acids linoleic acid and *α*-linolenic acid account for 2.10 and 0.71%, respectively; and the total contents of MUFA and PUFA are 25.5 and 2.81%, respectively (13).

Compared with edible beef tallow, camel hump oil has higher contents of MUFA, PUFA, linoleic acid, and α-linolenic acid, but a lower saturated fatty acid content. Additionally, hump tissue exhibits higher collagen content and significant levels of vitamin A (5.6 mg/kg) and vitamin E (19.4 mg/kg), the latter reflecting carotenoid precursors. In dromedary CHF, vitamin E levels have been reported as 0.003–0.00932% (≈30–93 mg/kg) (14) and 0.00522 g/100 g (≈52.2 mg/kg) (15), these data highlight its unique nutritional composition and potential functional value (16).

## 3 The three major value areas of camel hump: therapeutic, nutritional, and skin care

### 3.1 Medicinal and therapeutic value

Li Shizhen’s Compendium of Materia Medica (Ming Dynasty) notes (17, 18): “Camel fat refers to the hump. The fat within the hump is called hump oil. It has a sweet, warm taste and is non-toxic. It was historically believed to soften the five metals”. The Shengji Zonglu, a compilation of folk and medical prescriptions collected by the Northern Song court, records the “Apricot Kernel Paste Formula: Apricot kernels (five measures, raw) as the sole ingredient. Burn until smoke emerges, then extinguish and finely grind. Take two ounces of camel fat, melt and strain to remove membranes, mix evenly into a paste. Apply to the swelling and apply indirect heat with a candle. This treats wind-induced swelling” (19). In traditional Mongolian and Kazakh medicine, camel fat is used for skin injuries, rheumatic joint swelling and pain, and malunion of fractures (20). It should be noted that these historical descriptions are based on empirical observations and traditional beliefs, not on controlled clinical trials. Therefore, they should not be interpreted as evidence of efficacy in the modern medical sense.

Notably, relevant pharmacological evidence has been reported for purified oleic acid (OA), a major constituent of CHF, though direct evidence using intact CHF is currently lacking. Specifically, purified OA has been reported to exert antinociceptive, anti-inflammatory, and antioxidant effects in UVB-induced skin photodamage, a prevalent public health issue characterized by pain, edema, and erythema (21).

Using a mouse sunburn model challenged with 0.75 J/cm^2^ UVB radiation, Serafini et al. demonstrated that topical administration of OA (0.3–3%) resulted indose-dependent therapeutic benefits. Specifically, 3% OA completely abolished mechanical allodynia and thermal hypersensitivity, reduced affective pain responses by 72 ± 14%, alleviated paw edema by 58 ± 11%, and decreased polymorphonuclear leukocyte infiltration by 54 ± 10%. This treatment also effectively normalized serum reactive oxygen species (ROS) levels. Remarkably, the efficacy of 3% OA was statistically comparable to that of 0.1% dexamethasone across all measured parameters (22).

These findings suggest that purified OA acts as a multi-target molecule that simultaneously modulates nociceptive signaling, inflammatory cascades, and oxidative stress. These preclinical data for purified OA offer a rationale for exploring its potential in UVB-induced skin conditions; however, whether these effects translate to intact CHF, and whether they support traditional skin-related applications of CHF, requires direct experimental investigation meanwhile, camel humps possess specialized immune and endocrine functions along with osmoregulatory capabilities, which are crucial for their survival in harsh desert environments. Gene Ontology (GO) and Kyoto Encyclopedia of Genes and Genomes (KEGG) pathway analyses indicate that the identified differentially expressed genes (DEGs) reveal specialized immune and endocrine functions in camel humps, beyond osmoregulation. DEGs associated with human diseases were significantly upregulated in visceral fat (VF), while DEGs linked to antihypertensive and antidiabetic mechanisms were elevated in hump fat combined with subcutaneous fat (HS). Thus, HS appears more health-promoting than visceral fat. Simultaneously, the anterior hump exhibits greater immune and endocrine gene diversity than subcutaneous fat. Thus, the anterior hump may hold significant survival importance for Bactrian camels. Further exploration of other adaptive mechanisms requires acquiring additional genetic and molecular mechanism information (23).

Additionally, the proteome of the Bactrian camel hump was identified using proteomic sequencing technology, followed by GO annotation and KEGG pathway prediction via bioinformatics tools. A total of 1,077 proteins were identified, revealing that the Bactrian camel hump is rich in diverse functional proteins involved in cellular processes, metabolic processes, binding, catalytic activity, cells, cellular components, and organelles. KEGG analysis identified 301 distinct pathways within the humps. The majority of these pathways were associated with signal transduction, metabolic pathways, and energy metabolism. This proteome profile will contribute to elucidating the functional mechanisms of the Bactrian camel’s humps (24).

Guo et al. demonstrated that during fasting, insulin sensitivity decreased in the anterior hump and posterior hump, while muscle insulin sensitivity increased. However, overall, camels exhibited insulin resistance and increased lipolysis. Furthermore, serum levels of branched-chain amino acids (BCAAs) and aromatic amino acids (AAAs), along with the abundance of the phyla Firmicutes and Actinobacteria and the genera Bacteroides and Ruminococcus, serve as candidate biomarkers for diagnosing insulin resistance in camels; these findings provide direction for future research. Our findings reveal the molecular mechanisms underlying camelid hunger tolerance and may provide a novel animal model for human metabolic disease research (25).

Additionally, Hadipour et al. investigated the effects of replacing traditional rumen-protective fat sources, such as palm fatty acid powder, with CHF on the performance, carcass characteristics, blood biochemical parameters, and immune responses of finishing lambs. The results indicated that CHF is a suitable alternative to rumen-protected fat sources for finishing lambs and has positive effects on immune responses. (26).

### 3.2 Value in the food application sector

Camel humps were considered prized delicacies in ancient China. Zhou Mi’s Miscellaneous Notes from the Guixin Era (Song Dynasty) states: “The delicacy of camel humps ranks among the Eight Treasures”. Camel fat, particularly hump fat, is used in the preparation of various dishes across Asian and North African countries. CHF is utilized to produce cocoa butter substitutes (27). It is used to make high-quality semi-dry and dry sausages and for frying among Bedouin tribes in the Arabian Desert—due to its ability to withstand high temperatures without smoking. Additionally, it features in numerous Saudi Arabian dishes, such as albulgman, the most common staple food among desert dwellers (28).

When evaluating the resource potential of novel edible oils, their fatty acid composition and thermal stability are key indicators. Sbihi et al. demonstrated that Hachi fat, derived from camel humps, exhibits a distinctive composition rich in oleic and palmitic acids. This characteristic ratio not only suggests potential nutritional benefits but also confers favorable oxidative stability to the oil. Thermal analysis further confirms Hachi fat’s structural stability at elevated temperatures, meeting the demanding requirements of repeated home frying. These systematic characterization parameters collectively indicate that Hachi fat represents a promising natural solid fat resource. It provides a robust scientific basis for replacing traditional oils in the food industry, particularly for developing high-quality frying oils, and holds considerable potential for future commercial applications (29).

Belguith et al. conducted a pioneering study, successfully replicating authentic Tunisian traditional sun-dried merguez sausage for the first time using camel meat and hump fat as core ingredients. The study systematically tracked physicochemical and compositional changes during the sausage’s 12-day air-drying process. Results revealed that the dried product exhibited low water activity (0.673), low pH (4.97), and excellent microbiological quality, ensuring long-term stability at room temperature. This study confirms that using hump fat not only adheres to tradition but also endows camel merguez sausage with a superior fatty acid profile characterized by high oleic acid content. This provides a new pathway for the high-value utilization of such localized camel by-products (30, 31).

### 3.3 Application in skincare products

With sustainable and natural ingredients gaining prominence in the cosmetics sector, CHF has attracted research interest due to its unique fatty acid profile and bioactivity. Preliminary data suggest that CHF may possess biochemical properties relevant to cosmetic use, though robust evidence from *in vivo* and clinical studies remains limited.

Recent studies have reported the presence of essential fatty acids (e.g., omega-3, omega-6, and omega-9) as well as natural antioxidants such as tocopherols and carotenoids in CHF (see Section 2.2.4 Comparative Nutritional Properties of Camel Fat for detailed quantitative data) (32–34). It should be noted that these mechanisms were demonstrated for purified linoleic acid and tocopherols, not intact CHF. These constituents may contribute to skin health through multiple mechanisms. Mechanistically, purified linoleic acid (an omega-6 fatty acid present in CHF) has been reported to activate the PI3K/Akt signaling pathway in keratinocytes, potentially upregulating key enzymes in glutathione (GSH) biosynthesis. The resulting increase in GSH may enhance cellular redox capacity and potentially contribute to protection from oxidative damage. Moreover, purified linoleic acid has been reported to suppress UVB-induced COX-2 expression and prostaglandin E2 synthesis, reducing inflammation. Meanwhile, tocopherols and carotenoids act as direct radical scavengers. Collectively, these findings for purified constituents suggest that CHF may warrant further investigation as a natural ingredient for dermatological and cosmetic applications, pending direct *in vivo* and clinical validation (35).

According to GO and KEGG pathway analysis, pre-hump fat exhibits enrichment in more pathways and biological processes compared to post-hump and subcutaneous fat. The enriched pathways associated with the anterior hump primarily relate to the immune system, such as chemokine signaling pathways, Fc epsilon RI (FcεRI) signaling pathways, B cell receptor signaling pathways, Fc gamma R (FcγR)-mediated phagocytosis, and leukocyte transendothelial migration. Chemokine signaling pathways are considered to be closely related to health and aging (36).

The research by Boubal et al. systematically revealed the considerable potential of camel fat in the cosmetics industry. Its unique lipid composition features stable levels of saturated fatty acids (C16:0) at 33.5–34.0% and a prominent proportion of oleic acid (C18:1) at 40.3–41.8%. The study notably identified a pronounced “gender advantage”: female camels, particularly during the reproductive period, exhibited not only a more stable lipid profile but also enhanced antioxidant activity (DPPH scavenging rate up to 78%) and anti-aging enzyme activities. These results indicate the potential of CHF to mitigate oxidative stress. Anti-aging effects (such as anti-elastase, anti-tyrosinase, and anti-collagenase activities) were more pronounced in female samples. CHF, as a natural ingredient with significant bioactivity, represents a key component for developing natural cosmetics with promising efficacy through refined raw material selection based on gender and physiological state (37).

Australia and South Korea have launched pharmaceuticals, health supplements, beauty products, and moisturizing soaps produced using camel fat (hump fat, camel milk fat) as raw materials (38, 39). A research team in Inner Mongolia, China, has successfully developed hump refining technology and incorporated camel fat into the National List of Cosmetic Ingredients in Use.

In summary, the compositional profile and preliminary bioactivity data of CHF suggest it as a potential candidate for developing natural, sustainable, and non-toxic alternatives to synthetic ingredients commonly used in modern cosmetic formulations.

### 3.4 Critical analysis and limitations of current bioactivity and application research

Despite these promising applications, current evidence remains limited by several critical gaps. Most findings are derived from *in vitro* experiments or traditional empirical records, lacking rigorous *in vivo* validation, controlled clinical trials, and comprehensive toxicological evaluations. Inconsistencies in reported bioactivities—including antioxidant, anti-aging, and anti-inflammatory effects—are frequently observed, mainly due to variations in raw material sources, extraction procedures, and analytical protocols, along with the absence of standardized methods and limited sample representativeness.

Beyond biological evidence, substantial practical challenges hinder industrial translation. The relatively high unsaturated fatty acid content (approximately 40%) in CHF raises inherent concerns regarding oxidative stability during processing and storage, yet systematic stability data remain scarce. Furthermore, key quality control indicators—such as acid value, peroxide value, and fatty acid profiles—lack unified standards across studies. In addition, regulatory frameworks for CHF-derived food and cosmetic products remain insufficient or undefined in major markets, including the EU Novel Food catalogue, FDA GRAS status, and China’s cosmetic ingredient specifications, where purity criteria and safety thresholds have not been clearly established. Collectively, these biological, technical, and regulatory limitations collectively impede the reliable development and commercial translation of camel hump-based products.

## 4 Insights for bionics and materials science

The camel hump embodies three fundamental biomimetic principles: (i) spatial energy concentration (storing high-density fat in a confined anatomical volume), (ii) temporal energy buffering (accumulating energy during resource abundance and releasing it during scarcity), and (iii) dual-function insulation and regulation (simultaneous thermal protection via fat and thermohygroscopic management via sweat glands). Recent studies have translated these principles into synthetic materials and devices, as detailed below.

Short bowel syndrome (SBS) is characterized by markedly shortened intestinal transit time and severely impaired nutrient absorption, accompanied by excessive secretion of digestive fluids that further compromises nutritional status. Drawing inspiration from the camel hump’s unique ability to stably store energy reserves and release them gradually when needed, Dai et al. developed a hump-inspired ingestible magnetic capsule (IMC) for SBS treatment. This capsule comprises thermosensitive hydrogel microparticles based on poly(N-isopropylacrylamide) and acrylamide, along with a magnetic shell made of neodymium iron boron (NdFeB) and polyvinyl alcohol. This bionic design enables cyclic nutrient storage and sustained release, thereby improving enteral nutrition support for SBS patients. Overall, this work provides proof-of-concept for nutrient storage and controlled delivery using ingestible capsules, representing a promising therapeutic strategy for SBS (40). *Biomimetic mechanism:* The camel hump stores energy-dense fat in a confined space and releases it gradually. Mimicking this ‘spatially confined energy storage with controlled release’ principle, the IMC uses thermosensitive hydrogel microparticles (analogous to adipocytes) and a magnetic shell (analogous to the hump’s connective tissue capsule) for on-demand nutrient release.

Meanwhile, Liu et al. proposed a camel-hump-like adsorption strategy, utilizing ZIF nanoribbons to modulate ultralight self-supporting Na₄Mn₉O₁₈ films for embedding phase-change materials. This layered structure interconnected by Na₄Mn₉O₁₈ enhances photon trapping capabilities and provides a fast pathway for phonon transport. Notably, ZIF nanoribbons exhibit fat-storage functionality analogous to camel humps, enabling efficient accumulation and storage of solar energy (41). *Biomimetic mechanism:* The camel hump’s ability to store thermal energy and insulate against desert extremes inspired a ‘camel-hump-like adsorption strategy.’ ZIF nanoribbons enable solar-thermal capture (analogous to fat absorbing heat), while the Na₄Mn₉O₁₈ film provides insulation (analogous to connective tissue). The term ‘urban energy humps’ is now explicitly defined as a distributed thermal storage system that buffers energy supply – demand mismatches.

Additionally, camel humps achieve efficient thermal protection and thermohygroscopic management through internal fat tissue insulation combined with sweat gland regulation of perspiration flow. Inspired by this, Xu et al. developed a hump-inspired hierarchical fabric (HHF) using hot-rolling and ultrasonic welding techniques. This fabric ingeniously integrates an array of insulating units with shaped capillary channels, simultaneously delivering outstanding thermal protection and active thermal-humidity comfort management. It holds broad application prospects in fields such as protective clothing (42). *Biomimetic mechanism:* The hump’s thermal protection arises from two synergistic mechanisms: (i) subcutaneous fat as insulator, (ii) sweat gland evaporative cooling. The HHF emulates this dual system with insulating units (mimicking fat lobules) and capillary channels (mimicking sweat ducts).

Bionics has evolved from morphological mimicry to a deep analysis of biological systems’ material and energy management mechanisms. “Camel Hump Bionics” has thus distilled a core design philosophy for addressing energy temporal storage and spatial management: constructing a universal framework integrating “energy storage, thermal insulation, and regulation.” This framework is driving innovation across multiple domains: In smart energy, it guides the development of phase-change materials integrating solar-thermal conversion and thermal management, providing a blueprint for efficient “urban energy humps”; In biomedicine, it inspires the creation of miniature “artificial organs” that sense environments and intelligently release drugs, advancing treatment from “passive delivery” to “active management”; In personal thermal management, it has spawned active thermo-humidity control systems like HHF fabrics that dynamically respond to environments (43–45). To further clarify the biomimetic mapping, we present (Table 2) below, which links each synthetic material to the specific camel hump principle(s) it emulates.

**Table 2:** Functional principles of camel hump and their corresponding synthetic biomimetic materials.

Biomimetic principle	Camel hump feature	Synthetic material/device	Key analogous property
Spatial energy concentration	Fat stored in confined hump volume	IMC (Dai et al.)	Hydrogel microparticles as artificial adipocytes
Temporal energy buffering	Fat accumulation and mobilization	ZIF nanoribbons/PCM	Solar-thermal storage and release
Dual insulation + regulation	Fat insulation + sweat gland cooling	HHF fabric (Xu et al.)	Insulating units + capillary channels

Despite the broad application prospects of the above bionic strategies, relevant research remains largely at the proof-of-concept stage. Most studies focus on structural and functional simulation, lacking systematic verification of long-term stability, mechanical durability, and environmental adaptability. Methodological inconsistencies exist in material preparation processes, performance testing conditions, and evaluation indicators, leading to poor comparability across different studies. In addition, relevant research is mostly limited to laboratory-scale preparation, with insufficient attention to cost-effective, scalable manufacturing. The evidence supporting practical application remains preliminary, and further optimization and comprehensive performance evaluation are urgently needed.

## 5 Industrial application potential of camel humps

In the leather industry, CHF has shown potential as a renewable bio-based raw material for green fatliquoring agents. According to Habib, CHF can be chemically modified through sulfitation using sodium metabisulfite to produce a high-performance fatliquor for leather processing. The resulting fatliquor exhibits excellent emulsion stability against metal ions, tanning agents, and pH variations commonly encountered in leather manufacturing. Moreover, it effectively penetrates leather collagen fibres, forming a uniform lubricating layer that enhances the softness, fullness, and mechanical properties—such as tensile strength and elongation at break—of the finished leather. This approach not only enables the high-value utilization of CHF but also aligns with the sustainable development trend in the leather industry, which increasingly favours eco-friendly bio-based additives (46).

However, this preliminary study presents several notable limitations. The research was restricted to laboratory-scale preparation and performance evaluation, with no assessment of scalability, cost-effectiveness, or long-term stability for industrial production. Moreover, comprehensive safety and environmental risk assessments, including biodegradability, cytotoxicity, and potential residual risks, were lacking. In addition, the study did not deeply investigate the structure–activity relationship between CHF composition and fatliquoring properties, which restricts further optimization and large-scale industrial application of such bio-based fatliquoring agents.

## 6 Summary and outlook

Camel hump represents an underutilized animal-derived resource with distinctive biochemical composition, adaptive biological functions, and emerging multifunctional application potential. Current evidence indicates that CHF is characterized by high proportions of saturated and monounsaturated fatty acids, particularly palmitic, stearic, and oleic acids, together with functional minor components such as tocopherols, carotenoids, vitamins, and collagen-related constituents. These compositional features provide a scientific basis for its potential use in food processing, cosmetic formulation, dermatological care, biomimetic thermal management, and bio-based industrial materials. Notably, recent transcriptomic and proteomic studies suggest that the hump should not be regarded merely as a passive lipid depot, but rather as a metabolically active tissue involved in energy regulation, immune responses, and endocrine activity.

Despite these promising attributes, the available body of research remains fragmented and preliminary. Most studies are limited by small sample sizes, narrow breed or regional coverage, insufficient control of age, sex, diet, physiological status, and hump location, as well as non-standardized extraction and analytical protocols. These limitations have contributed to inconsistencies in reported fatty acid profiles, physicochemical properties, antioxidant activities, and anti-aging or anti-inflammatory effects. In addition, many functional claims are still based on *in vitro* assays, traditional empirical records, or extrapolation from individual components such as oleic acid and linoleic acid. Rigorous *in vivo* validation, toxicological assessment, dose–response analysis, and well-designed clinical or application-oriented studies are still lacking. Therefore, the biological efficacy and safety of camel hump-derived ingredients should be interpreted with caution until stronger experimental evidence becomes available.

Future research should prioritize establishing standardized raw material and quality control systems. Key variables, including camel species, breed, sex, age, feeding pattern, physiological stage, anatomical sampling site, storage condition, and extraction method, should be clearly reported and systematically compared. Unified analytical standards for fatty acid composition, acid value, peroxide value, iodine value, cholesterol level, oxidative stability, and bioactive minor constituents are essential for improving reproducibility and enabling cross-study comparison. For food and cosmetic applications, special attention should be paid to oxidative stability, sensory properties, allergenic risk, microbiological safety, formulation compatibility, and regulatory compliance. These efforts will be critical for transforming CHF from a traditional regional resource into a reliable and controllable industrial ingredient.

Mechanistic studies also require further strengthening. Integrated multi-omics approaches, including genomics, transcriptomics, proteomics, lipidomics, metabolomics, and microbiome analysis, may help clarify the regulatory networks underlying lipid storage, fasting tolerance, immune modulation, endocrine activity, and environmental adaptation in camels. Such studies could not only deepen the biological understanding of camel hump function, but also provide new insights into metabolic regulation, stress adaptation, and potential translational models for human health research. In parallel, studies on functional components should move from descriptive compositional analysis toward target identification, pathway verification, bioavailability assessment, and structure–activity relationship clarification.

From an application perspective, camel hump-derived materials hold promise in several sustainable development scenarios. In the food industry, CHF may contribute to the development of regionally distinctive meat products, frying fats, and structured lipid systems, provided that nutritional value, processing stability, and consumer acceptance are systematically evaluated. In cosmetics and dermatological formulations, its lipid profile and antioxidant potential support further exploration as a natural emollient or functional ingredient, but efficacy and safety must be validated using standardized models and human-relevant testing systems. In materials science, camel hump-inspired designs have already stimulated innovative concepts in controlled nutrient delivery, solar-thermal energy storage, and personal thermal management textiles. However, these biomimetic systems remain largely at the proof-of-concept stage and require further optimization in durability, scalability, cost-effectiveness, and real-world performance.

Overall, the high-value utilization of camel hump requires a transition from scattered compositional and empirical studies toward standardized, mechanism-driven, and application-oriented research. By integrating animal science, food chemistry, pharmacology, dermatology, materials engineering, and industrial biotechnology, future studies can build a more robust scientific foundation for camel hump-derived products. Such efforts may promote the sustainable use of camel resources, support circular bioeconomy development in arid and semi-arid regions, and offer new bio-based solutions for food, health, cosmetics, and advanced materials industries.
