# Supplementary material for: Camel hump: composition, bioactivities, and multifunctional sustainable applications
Source: Front Nutr. 2026 Jun 25;13:1869754. doi: 10.3389/fnut.2026.1869754 (PMC13346034; doi:10.3389/fnut.2026.1869754)
Supplement: Supplementary file 1 [file Supplementary_File_1.docx]

**Supplementary File — Methodological Details: Experimental Protocols for Camel Hump Fat Analysis in Cited Studies**

Note to reviewers: The present work is a review article and does not report primary experimental data. The detailed experimental parameters (extraction temperatures, specific reagents, GC‑MS conditions, etc.) required for reproducibility are the responsibility of the original primary studies cited herein. The following table compiles the key methodological information and compositional data extracted from those original sources to assist readers in assessing data comparability across studies. Parameter fields marked “NR” indicate “not reported” in the original publication.

| Table S1. Methodological summary of key original studies cited in the review | | | | | | | | | | |
| --- | --- | --- | --- | --- | --- | --- | --- | --- | --- | --- |
| Study (Ref.) | Species | Sample origin / age | Hump location | Extraction method | Solvent system | Extraction temp / time | Fatty acid analysis method | Methylation method | GC column | Key data reported |
| Wu Yun (Master's thesis, 2013) [10] | Bactrian (Camelus bactrianus) | Inner Mongolia, China; n=27 (5–7 y, 8–14 y, both sexes and castrates) | Fore hump & rear hump (separately) | Soxhlet extraction | Petroleum ether (boiling point 60–90 °C) | ~75 °C / 8–10 h | GC‑MS | BF₃‑methanol (14%, w/v), 60 °C, 30 min | DB‑23 (60 m × 0.25 mm × 0.25 µm) | Fatty acid profile (15 FA identified), iodine value, acid value, saponification value, moisture, protein, crude fat, cholesterol (42.33 mg/100g), vitamin A (0.56 mg/100g), vitamin E (1.94 mg/100g) with means ± SD |
| Wu Yun et al. (China Oils and Fats, 2013) [9] | Bactrian (Camelus bactrianus) | Alxa League, Inner Mongolia | Fore & rear hump (pooled) | Soxhlet extraction | Petroleum ether | ~75 °C / 8–10 h (inferred) | GC‑MS | BF₃‑methanol (14%), 60 °C, 30 min (inferred) | DB‑23 (60 m) (inferred) | Fatty acid composition (14 FA identified, crude fat 90.78%); SFA 56.39%, UFA 37.29%, C18:1 31.03%, C18:0 26.48%, C16:0 21.85% |
| Liu Donghui (Master's thesis, 2017) [3] | Bactrian (Camelus bactrianus) | Urad Rear Banner, Inner Mongolia; n=3 per fat location (fore hump, rear hump, subcutaneous, milk fat) | Fore hump, rear hump, subcutaneous, milk fat | Hot‑press filtration & alkali refining | None (physical pressing + chemical refining) | ~60 °C (melting) + 25 min emulsification | GC‑MS | BF₃‑methanol (14%), 80 °C, 2 min (inferred) | SP‑2560 (100 m × 0.25 mm × 0.20 µm) | Fatty acid profile (20 FA identified), acid value, iodine value, saponification value, peroxide value with means ± SD; soft‑making process details (emulsification temperature 75 °C, homogenization 2000 rpm, etc.) |
| El‑Anany et al. (Riv. Ital. Sostanze Grasse, 2018) [5] | Dromedary (Camelus dromedarius) | Egypt (exact region NR) | NR (hump fat) | NR | NR | NR | GC | NR | NR | Fatty acid composition (SFA 63.40%, MUFA 34.68%, PUFA 1.92%), melting point (78 °C), iodine value (63.50 g I₂/100g), acid value (0.99 mg KOH/g), saponification value (203.1 mg KOH/g) |
| Sahraoui et al. (J. Camel Pract. Res., 2015) [7] | Dromedary (Camelus dromedarius) | Algeria; n=43 (1–13 y, both sexes, Sahraoui & Tergui breeds) | NR (hump fat) | NR | NR | NR | GC | NR | NR | Fatty acid composition (SFA 64.4%, MUFA 33.1%, PUFA 2.5%, C16:0 31.5%, C18:0 25.5%, C18:1 25.9%, C18:2 1.17%, C18:3 0.42%) |

Abbreviations: FA, fatty acids; NR, not reported in original publication; SFA, saturated fatty acids; MUFA, monounsaturated fatty acids; PUFA, polyunsaturated fatty acids; UFA, unsaturated fatty acids; GC‑MS, gas chromatography‑mass spectrometry; BF₃, boron trifluoride.

| Table S2. Key compositional data extracted from the five original studies | | | | | |
| --- | --- | --- | --- | --- | --- |
| Parameter | Wu Yun (2013,Master’s) | Wu Yun et al.  (2013, China Oils Fats) | Liu Donghui  (2017, Master’s) | El‑Anany et al. (2018) | Sahraoui et al. (2015) |
| Species | Bactrian | Bactrian | Bactrian | Dromedary | Dromedary |
| Hump location | Fore & rear (separate) | Fore & rear (pooled) | Fore, rear, subcutaneous, milk | NR | NR |
| Fatty acids (%, of total FA) |  |  |  |  |  |
| C14:0 | 6.41–7.59 | NR | 6.41–7.59 | NR | NR |
| C16:0 | 33.57–35.11 | 21.85 | 33.57–35.11 | Not isolated (blended) | 31.5 |
| C18:0 | 19.58–22.18 | 26.48 | 19.58–22.18 | Not isolated (blended) | 25.5 |
| C18:1 (oleic) | 25.04–28.58 | 31.03 | 25.04–28.58 | Not isolated (blended) | 25.9 |
| C18:2 (linoleic) | 1.92–2.19 | NR | 1.92–2.19 | Not isolated (blended) | 1.17 |
| C18:3 (α‑linolenic) | 1.13–1.22 | NR | 1.13–1.22 | Not isolated (blended) | 0.42 |
| SFA | 63.63–65.9 | 56.39 | 62.64–65.9 | 63.4 | 64.4 |
| MUFA | 30.23–33.27 | — | 30.23–33.27 | 34.68 | 33.1 |
| PUFA | 3.22–3.59 | — | 3.15–3.68 | 1.92 | 2.5 |
| Physicochemical properties |  |  |  |  |  |
| Acid value (mg KOH/g) | 0.48–0.77 | NR | 0.54 (tissue) / 6.82 (milk) | 0.99 | NR |
| Iodine value (g I₂/100g) | 36.67–39.05 | NR | 44.53 (tissue) / 34.00 (milk) | 63.5 | NR |
| Saponification value (mg KOH/g) | 198.73–205.36 | NR | 200.45 (tissue) / 212.60 (milk) | 203.1 | NR |
| Peroxide value (mmol/kg) | 2.37–2.41 | NR | 2.39 (tissue) / 1.16 (milk) | NR | NR |
| Melting point (°C) | 46.08–51.54 | NR | NR (softening ~60 °C) | 78 | NR |
| Other components |  |  |  |  |  |
| Crude fat (%) | 88.20–93.19 | 90.78 | 88.20–93.19 (tissue) | NR | NR |
| Moisture (%) | 5.68–9.21 | NR | 5.68–9.21 (tissue) | NR | NR |
| Protein (%) | 2.75–3.20 | NR | 2.75–3.20 (tissue) | NR | NR |
| Ash (%) | 0.164–0.231 | NR | 0.164–0.231 (tissue) | NR | NR |
| Cholesterol (mg/100g) | 42.33 | NR | 41.10–42.91 | NR | NR |
| Vitamin A (mg/100g) | 0.56 | NR | NR | NR | NR |
| Vitamin E (mg/100g) | 1.94 | NR | NR | NR | NR |

Note: Ranges indicate variation across fore hump vs. rear humAdditional method notes from the original studies

**Additional method notes from the original studies**

**Wu Yun (2013, Master’s thesis) — Supplemental method details:**

**Animal sampling:** 27 Alxa Bactrian camels (16 castrates, 11 females), including 8 young camels (5–7 years) and 19 adult camels (8–14 years). Fore hump and rear hump were collected separately from each animal.

**Fat extraction:** Soxhlet extraction using petroleum ether (boiling point 60–90 °C) at approximately 75 °C for 8–10 hours.

**Fatty acid methylation:** BF₃‑methanol solution (14%, w/v) at 60 °C for 30 minutes.

**GC‑MS conditions:** DB‑23 capillary column (60 m × 0.25 mm × 0.25 µm). Injector temperature 270 °C, ion source temperature 230 °C, quadrupole temperature 150 °C. Carrier gas: high‑purity helium at 1.0 mL/min. Split ratio 5:1. Oven temperature program: 130 °C held 10 min; ramp 6.5 °C/min to 170 °C; ramp 2.75 °C/min to 215 °C, held 12 min; ramp 40 °C/min to 230 °C, held 3 min.

**Fatty acid identification:** By comparison with Supelco 37 Component FAME Mix standards and CLA standard, using NIST05 mass spectral library.

**Cholesterol determination:** By GC using DB‑23 column (0.25 mm × 60 m × 0.25 µm). Injector 280 °C, detector 290 °C. Air flow 350 mL/min.

**Temperature program:** 200 °C held 1 min; ramp 30 °C/min to 280 °C, held 10 min. External standard quantification.

**Statistical analysis:**One‑way ANOVA with Duncan’s multiple range test using SPSS 22.0; significance at α = 0.05 and 0.01.

**Liu Donghui (2017, Master’s thesis) — Supplemental method details:**

**Fat extraction:** Tissue fat was melted at 60 °C, press‑filtered, dehydrated with anhydrous Na₂SO₄, deodorized by rotary evaporation at 60 °C, and alkali‑refined for decolorization. Milk fat: extracted by chloroform‑methanol method (45 °C water bath, 15 min).

**Fatty acid methylation:** BF₃‑methanol (14%) at 80 °C for 2 minutes (condensation reflux).

GC‑MS conditions: SP‑2560 capillary column (100 m × 0.25 mm × 0.20 µm). Injector temperature 250 °C, split ratio 10:1; FID detector at 280 °C; helium flow 0.9 mL/min. Oven program: 130 °C, ramp 4 °C/min to 230 °C, held 30 min.

**Physicochemical methods:** Acid value (GB/T 5530‑2005, hot ethanol method); iodine value (GB/T 5532‑2008, Hanus method); saponification value (GB/T 5534‑2008); peroxide value (GB/T 5538‑2005).

**Ointment preparation:** O/W emulsion. Oil phase: 20 g camel fat, 3 g cetyl alcohol, 2 g silicone oil, 0.1 g sea buckthorn oil, 2 g glycerol monostearate.

**Water phase:** 3.5 g Tween‑80, 3 g glycerol, 2 g propylene glycol, deionized water. Emulsification at 75 °C for 25 min with homogenization at 2000 rpm.

**El‑Anany et al. (2018) — Supplemental note:**

The original publication does not report extraction solvents, methylation conditions, or GC column details. The fatty acid composition data (SFA 63.40%, MUFA 34.68%, PUFA 1.92%) and physicochemical constants (melting point 78.0 °C, iodine value 63.50 g I₂/100g, saponification value 203.1 mg KOH/g, acid value 0.99 mg KOH/g) were taken as reported.

**Sahraoui et al. (2015) — Supplemental note:**

The original publication does not report the specific GC column, methylation protocol, or extraction method. The study analyzed hump fat from 43 Algerian dromedaries (1–13 years, both sexes, Sahraoui and Tergui breeds) and reported fatty acid composition on a weight percentage basis. The original data did not include standard deviations for the individual fatty acids; only breed‑level mean comparisons were provided. Therefore, the values in Table 1 and Table S2 are presented as reported mean values.

**Key take‑home points for the reviewer**

The five primary studies exhibit substantial methodological heterogeneity in extraction methods (Soxhlet with petroleum ether vs. hot‑press filtration vs. solvent extraction of milk fat), methylation conditions (BF₃‑methanol at 60 °C vs. 80 °C, 30 min vs. 2 min), and GC‑MS parameters (DB‑23 vs. SP‑2560 columns, different temperature programs). These differences — rather than true biological variation — may partly explain discrepancies in reported fatty acid values (e.g., oleic acid ranging from 25.04 % to 31.03 %) across studies.

Direct quantitative comparisons across studies should be made with caution, especially given that El‑Anany et al. (2018) and Sahraoui et al. (2015) did not report complete methodological parameters, making it difficult to assess comparability.

The review has added a critical discussion in Section 2.3 highlighting these methodological limitations, and the present Supplementary File provides a transparent compilation of what is and is not reported in each original source.

This Supplementary File is intended to assist readers in evaluating data comparability across primary studies. All experimental reproducibility details remain the responsibility of the original cited publications.
